# Phytochemistry, Mode of Action Predictions, and Synergistic Potential of *Hypenia irregularis* Essential Oil Mixtures for Controlling *Aedes aegypti*

**DOI:** 10.3390/toxins17080402

**Published:** 2025-08-11

**Authors:** Luis O. Viteri Jumbo, Wellington S. Moura, Richard D. Possel, Osmany M. Herrera, Rodrigo R. Fidelis, Bruno S. Andrade, Guy Smagghe, Gil R. Santos, Eugênio E. Oliveira, Raimundo W. S. Aguiar

**Affiliations:** 1 Programa de Pós-Graduação em Biotecnologia, Universidade Federal do Tocantins (UFT), Gurupi 77402-970, TO, Brazil; luis.viteri@uft.edu.br (L.O.V.J.); rikee.dias@hotmail.com (R.D.P.); bandrade@uesb.edu.br (B.S.A.); guysma9@gmail.com (G.S.); gilrsan@uft.edu.br (G.R.S.); 2 Programa de Pós-Graduação em Produção Vegetal, Universidade Federal de Tocantins (UFT), Gurupi 77402-970, TO, Brazil; osmany.herrera@uft.edu.br (O.M.H.); fidelisrr@mail.uft.edu.br (R.R.F.); 3 Programa de Pós-Graduação em Biologia Animal, Universidade Federal de Viçosa, Viçosa 36570-900, MG, Brazil; 4 Coordenação de Tecnologia em Segurança Pública, Universidade Estadual do Tocantins, Palmas 77020-122, TO, Brazil; wellington.sm@unitins.br; 5 Departmento de Ciencias Biológicas, Universidade Estadual do Sudoeste da Bahia, Jequié 45208-091, BA, Brazil; 6 Institute of Entomology, Guizhou University, Guiyang 550025, China; 7 Department of Biology, Vrije Universiteit Brussel (VUB), 1050 Brussels, Belgium; 8 Departamento de Entomologia, Universidade Federal de Viçosa, Viçosa 36570-900, MG, Brazil

**Keywords:** plant-based biorational insecticides, deterrence oviposition, molecular docking, biorational repellents

## Abstract

*Aedes aegypti*, also known as the yellow fever mosquito, presents a major public health challenge, highlighting the need for effective biorational agents for mosquito control. Here, we investigated the synergistic effects of essential oil mixtures derived from *Hypenia irregularis* that is a mint-family shrub native to Brazil’s Cerrado biome, known as “alecrim do Cerrado”, in combination with essential oils from noni (*Morinda citrifolia*), Brazilian mint (“salva-do-Marajó”, *Hyptis crenata*), and lemongrass (*Cymbopogon citratus*) against *Ae. aegypti*. We conducted phytochemical analyses and assessed larvicidal, repellent, and oviposition deterrent activities. Using in silico methods, we predicted molecular interactions between key essential oil components and physiological targets involved in repellent action (odorant-binding protein AeagOBP1 and olfactory receptor Or31) and larvicidal activity (GABA and octopamine receptors, TRP channels, and acetylcholinesterase [AChE]). Major compounds identified included octanoic acid (23%; *Hipe. irregularis* × *M. citrifolia*), 2,5-dimethoxy-*p*-cymene (21.9%; *Hipe. irregularis* × *Hypt. crenata*), and citral (23.0%; *Hipe. irregularis* × *C. citratus*). Although individual oils showed strong larvicidal activity (*Hipe. irregularis* LC_50_ = 2.35 µL/mL; *Hypt. crenata* = 2.37 µL/mL; *M. citrifolia* and *C. citratus* = 2.71 µL/mL), their mixtures did not display synergistic effects. Similarly, repellency and oviposition deterrence were comparable to DEET for individual oils but were not enhanced in mixtures. Notably, the *Hipe. irregularis* × *C. citratus* essential oil blend reduced oviposition deterrence. Molecular docking confirmed strong binding of major oil components to AeagOBP1 and Or31, supporting their role in repellency. For larvicidal effects, AChE showed the highest predicted binding affinity. Overall, our findings suggest that *H. irregularis*, *Hypt. crenata*, *C. citratus*, and *M. citrifolia* (alone or in 1:1 mixture) are promising, sustainable agents for *A. aegypti* control.

## 1. Introduction

The yellow fever mosquito, *Aedes aegypti* (Diptera: Culicidae) (Linnaeus, 1762), is a primary vector responsible for the transmission of several arboviruses, including dengue, yellow fever, chikungunya, and Zika. Consequently, it poses significant public health concerns. These vectors contribute to greater human morbidity and mortality than any other arthropod-borne viral diseases in tropical and subtropical regions worldwide [1]. In 2022, the World Health Organization (WHO) estimated approximately 390 million annual dengue virus infections globally, with 70% occurring in Asia [1]. In particular, Brazil reported nearly 3 million dengue cases in 2023 [2]. As of February 2024, over 512,000 cases had already been recorded—three times the number reported during the same period in 2023 [3]. These data underscore the urgent need for improved control strategies targeting *A. aegypti*, especially in Brazil, where its impact on public health is especially critical.

*Aedes aegypti* mosquitoes develop in a wide range of natural and artificial containers during their aquatic immature stages. As adults, females require blood meals to complete their reproductive cycle. If infected with a virus, they can transmit pathogens responsible for the aforementioned diseases through biting, resulting in thousands of human deaths annually [1,4]. As a preventive measure, public health authorities often apply synthetic insecticides in open areas and residential settings. However, extensive use of these chemical agents has raised environmental concerns, including harmful effects on non-target organisms and mammals [5,6]. Notably, N,N-diethyl-meta-toluamide (DEET) and its derivatives—widely used mosquito repellents for over 70 years—are associated with such adverse effects [6]. Furthermore, the growing resistance of *A. aegypti* populations to conventional insecticides presents a major challenge [7,8]. These issues have intensified interest in plant-derived products with biological activity against mosquito vectors, particularly essential oils, which have demonstrated both toxic effects and behavioral disruption in *Ae. aegypti*.

In this context, essential oils have emerged as promising agents with toxic effects against both adult and larval stages of mosquitoes, while offering the advantage of selectivity toward natural enemies [9,10,11,12]. Their chemical complexity, consisting of diverse molecular constituents, reduces the likelihood of resistance development in insect populations. Pavela and Benelli [13] noted that the biological efficacy of essential oils may arise from synergistic interactions among all their components or from the activity of major compounds present at higher concentrations. As a result, essential oils can act in multifaceted ways on various targets within the insect organism. Several studies have shown that these biomolecules interfere with key physiological and biochemical processes, including the inhibition or modulation of cytochrome P450 enzymes, acetylcholinesterase (AChE), glutathione S-transferase (GST), γ-aminobutyric acid (GABA) receptors, transient receptor potential (TRP) channels, and the cholinergic and octopaminergic systems [13,14,15,16,17,18,19,20,21,22]. Furthermore, essential oils are considered safe for human use and are already widely employed in pharmaceutical, food, cosmetic, and perfumery industries, as well as in aromatherapy and natural medicine [23].

The plants *Hypenia irregularis* (Benth.) Harley (Lamiaceae), *Morinda citrifolia* L. (Rubiaceae), *Hyptis crenata* Pohl ex Benth (Lamiaceae), and *Cymbopogon citratus* (DC.) Stapf (Poaceae) have demonstrated notable insecticidal, fungicidal, and acaricidal properties [24,25,26,27,28,29]. Moreover, the major constituents of their essential oils have been linked to behavioral and physiological changes in insects, including oviposition deterrence, antifeedant activity, and other behavioral modifications [30,31,32,33,34]. The distribution of these plants reflects a remarkable adaptation to diverse habitats and soil-climatic conditions [35,36,37,38]. While *Hype. irregularis* is a mint-family shrub mint native to the Central Brazilian Cerrado, where acidic, nutrient-poor soils and a seasonally dry tropical climate contribute to its unique secondary metabolism [35], *Hypt. crenata* plants, also known as Brazilian mint (“salva-do-Marajó”) occurs in the Amazon, especially near the Amazon River and Marajó Island, thriving in humid, sandy soils with variable rainfall and high temperatures, which shape its essential oil profile [36]. *Morinda citrifolia* grows widely across tropical regions from Southeast Asia to the Caribbean, tolerating diverse soils and climates, including drought and salinity [37]. *Cymbopogon citratus*, i.e., lemongrass, plants are cultivated globally in tropical areas, favoring well-drained, moderately fertile soil and warm, sunny conditions with high rainfall, but is sensitive to frost [38].

Oviposition deterrence, antifeedant activity, and other behavioral modifications effects mediated by plant-based essential oil are particularly relevant in vector species and should be incorporated into *A. aegypti* management strategies. For instance, repellency minimizes human–vector contact, a function typically targeted by commercial chemical repellents. In addition, oviposition deterrence can reduce egg density in high-risk areas, while oviposition attraction may be leveraged in attractant-baited traps as part of integrated control strategies. Therefore, both effects warrant further exploration. Although numerous studies have documented the efficacy of essential oils against mosquito vectors and attributed these effects to the synergistic actions of their chemical constituents [7,39], relatively few investigations have explored the potentially enhanced effects of combining two different essential oils.

Plant-derived green products are increasingly recognized as safe alternatives for vector control, acting either as preventive agents (e.g., repellents and oviposition deterrents) or as curative agents through their toxic effects, especially in vulnerable environments such as residential areas. Given the documented biological activity of *Hype. irregularis*, *M. citrifolia*, *Hypt. crenata*, and *C. citratus*, along with their natural availability in Brazil, these species represent a potentially sustainable source of biorational products that can synergistically affect *A. aegypti*.

Here, considering that the individual insecticidal potential of essential oils from *Hype. irregularis*, *M. citrifolia*, *Hypt. crenata*, and *C. citratus* has already been reported, we aimed to investigate whether *Hype. irregularis* essential oil exhibits synergistic effects against *A. aegypti* when combined with the essential oils of the other three plant species. Larvicidal activity (lethality), repellency, and oviposition deterrence were evaluated to compare the efficacy of *Hype. irregularis* essential oil alone and in combination with the others. Additionally, we conducted in silico predictions to explore the potential involvement of TRP channels, AChE, GABA and octopamine receptors in the toxicity of the major compounds identified in the essential oil mixtures. We also evaluated molecular targets related to repellency (e.g., odorant-binding protein AeagOBP1 and olfactory receptor Or31). These targets, which are structurally characterized in *A. aegypti* and associated with major classes of insecticides and repellents, were selected to provide preliminary insights into the potential modes of action of the essential oil combinations.

## 2. Results

### 2.1. Identification of Peaks in GC–MS of Essential Oils

Our results showed an essential oil extraction yield of 0.37% for *Hipe. irregularis*; of 2.98% for *M. citrifolia*; of 0.92% for *Hypt. crenata* and 0.73% for *C. citratus*. The phytochemical profiles of each essential oil were already described in previous investigations (Supplementary Table S1). Here, we our phytochemistry analysis revealed the octanoic acid (23.0%), 2,5-dimethoxy-*p*-cymene (19.5%), and α-cymene (10.0%) as the major constituents identified in the combination of *Hipe. irregularis* and *M. citrifolia* essential oils (Table 1). In the mixture of *Hipe. irregularis* with *Hypt. crenata*, the predominant compounds were 2,5-dimethoxy-*p*-cymene (21.9%), carvacrol (10.4%), and α-cymene (10.0%) (Table 1). For the combination of *Hipe. irregularis* and *C. citratus*, the most abundant components were citral A (23.0%), 2,5-dimethoxy-*p*-cymene (15.5%), and citral B (17.1%) (Table 1).

### 2.2. Toxicity of Essential Oils to Aedes aegypti Third-Instar Larvae

When tested individually, *Hipe. irregularis* essential oil exhibited similar larvicidal potential compared to the essential oils of the other plants (Table 2). When evaluating potential synergistic effects, only numerical variations were recorded among the different combinations and proportions, without presenting any statistical difference (Table 3).

### 2.3. Molecular Modeling Predictions

The selected templates for homology modeling are presented in Supplementary Table S2, along with their sequence identities, Ramachandran favored values, and QMEAN scores used for model validation. The templates selected for AChE, GABA receptor, TRP channel, Octopamine receptor, odorant-binding protein (OBP), and Odorant Receptor AaOr31 were XP_021699617.1, AAA68961.1, AAEL005437, XP_021692997.1, AaegOBP1, and AAEL013217, respectively (Supplementary Table S2).

The major compounds from the different plants were docked with various receptors, forming multiple types of interactions with varying binding affinities, as indicated by the docking assays (Table 4).

**Figure 1:**
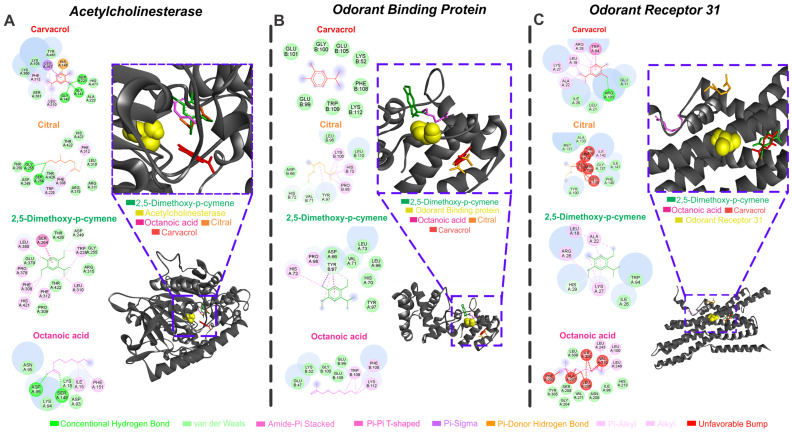
Carvacrol, citral, 2,5-dimethoxy-*p*-cymene, and octanoic acid bind with acetylcholinesterase (AChE) (**A**), Odorant Binding Protein (OBP) (**B**), and odorant receptor 31 (OR31) (**C**) target complexes of *Aedes aegypti*; the 2D maps of molecular interactions with amino acids in each target active site (yellow) are also shown.

The best affinity energy results were observed for AChE, OBP, and odorant receptor AaOr31. The molecular interactions of the essential oil major compounds with the active sites of three neurophysiological targets, i.e., AChE, odorant-binding protein (OBP), and odorant receptor 31 (OR31), revealed distinct binding profiles (Figure 1). Within the AChE active site, carvacrol exhibited strong hydrophobic interactions, including pi–alkyl contacts with TYR341 and PHE312 and a pi–pi stacking interaction with TRP86, although an unfavorable steric clash was noted with HIS438. Citral formed multiple hydrophobic contacts, including pi–alkyl, pi–sigma, and alkyl interactions, primarily with THR250, PHE295, ALA310, and TRP425, suggesting a relatively flexible accommodation within the catalytic pocket. 2,5-dimethoxy-*p*-cymene displayed conventional hydrogen bonding with SER254 and ARG255, along with van der Waals forces involving GLY258, LEU310, and GLU379, contributing to the stability of the ligand–protein complex. Octanoic acid engaged mainly in conventional hydrogen bonds with ASP74 and ASN96, as well as van der Waals interactions with LYS75, ILE95, and PHE151, indicating a more polar interaction profile. Regarding OBP, carvacrol established amide pi-stacked and pi–alkyl interactions with aromatic residues such as TRP109, PHE110, and GLU101, stabilizing the ligand within the binding pocket. 

Citral engaged in a network of pi–donor hydrogen bonds and pi–alkyl contacts involving ASP66, VAL67, LYS110, and TYR97, supporting moderate affinity and a flexible orientation. 2,5-dimethoxy-*p*-cymene formed hydrogen bonds with ASP96 and TYR97, along with van der Waals interactions involving HIS70, PRO98, and LEU73, reflecting a balanced hydrophobic and polar interaction pattern.

Octanoic acid exhibited alkyl, pi–alkyl, and pi–sigma interactions with GLU105, VAL100, and LYS112, anchoring the ligand within the hydrophobic core of the protein. In the OR31 binding domain, carvacrol formed pi–alkyl interactions with ARG23 and TRP49, while also presenting an unfavorable steric clash near LYS24, potentially compromising binding stability. Citral primarily engaged in hydrophobic interactions, including alkyl and pi–alkyl contacts with MET131, PHE134, ILE141, and ALA133, suggesting tight packing within the transmembrane region. 2,5-dimethoxy-*p*-cymene displayed hydrogen bonding with HIS25 and ARG26 and van der Waals interactions with LEU18, TRP94, and LYS27, indicating a strong and stable binding configuration. Finally, octanoic acid interacted through alkyl and van der Waals contacts with ILE43, ILE45, SER109, and PHE108, consistent with a predominantly hydrophobic binding mode.

Additionally, molecular interactions with the GABA receptor, TRP channel, and Octopamine receptor, targets that exhibited lower binding affinities, are also shown Supplementary Materials (Figure S1 and corresponding text).

### 2.4. Repellence of Essential Oils Against Aedes aegypti Adults

**Figure 2:**
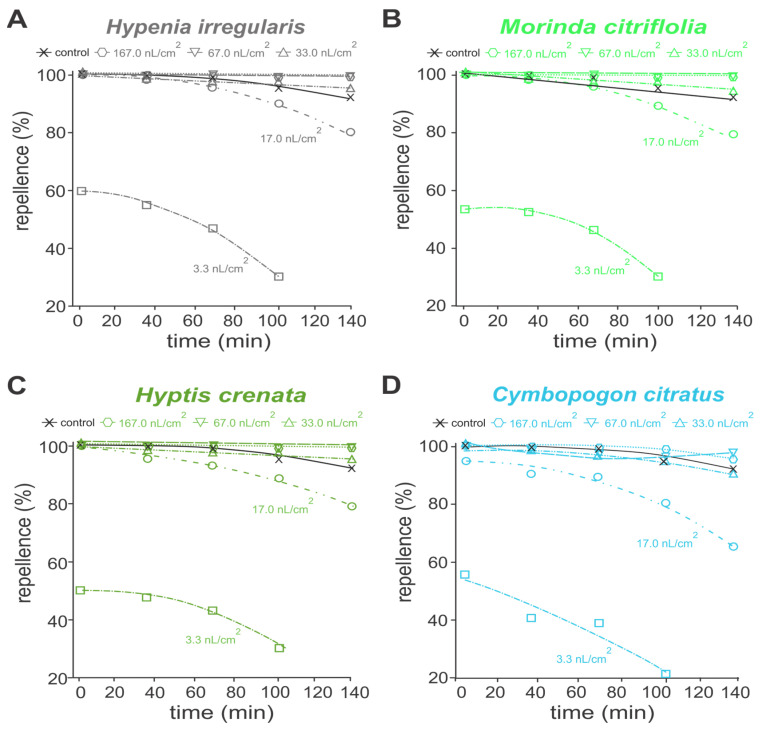
Protectant activity over time of different concentrations of essential oils of *Hypenia irregularis* (**A**); *Morinda citrifolia* (**B**); *Hyptis crenata* (**C**); and *Cymbopogon citratus* (**D**) against adults of *Aedes aegypti*. (**A**–**D**) Control refers to a commercial formulation containing DEET (15%).

**Figure 3:**
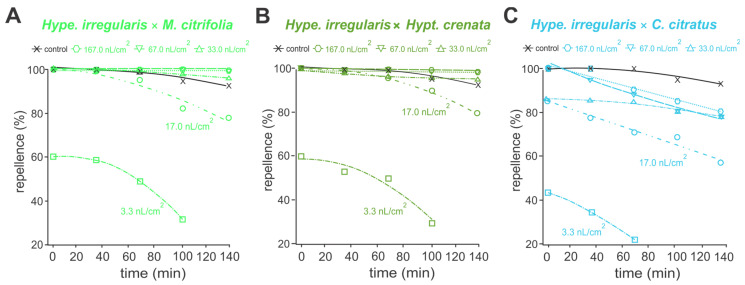
Protectant activity against adults of *Aedes aegypti* of a mixture (1:1) of essential oils of *Hypenia irregularis* × *Morinda citrifolia* (**A**), *Hypenia irregularis* × *Hyptis crenata* (**B**) and *Hipe. irregularis* × *Cymbopogon citratus* (**C**) at different concentrations. (**A**–**C**) Control refers to a commercial formulation containing DEET (15%).

In the analysis of protective activity, essential oils of *Hipe. Irregularis*, *M. citrifolia* and *Hypt. crenata* repelled at least 80% of *A. aegypti* adults, for up to 140 mines, at concentrations above 17.0 nL/cm (Figure 2A–C). Essential oil of *C. citratus* retained such repellent actions only up to 100 min (Figure 2D). When pure essential oils were applied at concentrations above 33.0 nL/cm^2^, the repellent performances were similar to the commercial repellent DEET (15%), providing more than 95% protection (Figure 2). Similar oviposition deterrence performances were reported to the 1:1 essential oil mixtures, except for those containing *C. citratus* essential oil (Figure 3). Mixtures of *Hipe. irregularis* with either *M. citrifolia* or *Hypt. crenata* at concentrations above 17.0 nL/cm^2^ repelled more than 80% of *A. aegypti* adults for up to 140 min (Figure 3A–B). In contrast, the repellent effect of the *Hipe. irregularis* and *C. citratus* mixture declined over time across all tested concentrations (Figure 3C), suggesting an antagonistic interaction.

### 2.5. Oviposition Deterrence Effects in Aedes aegypti Mediated by Essential Oils

**Figure 4:**
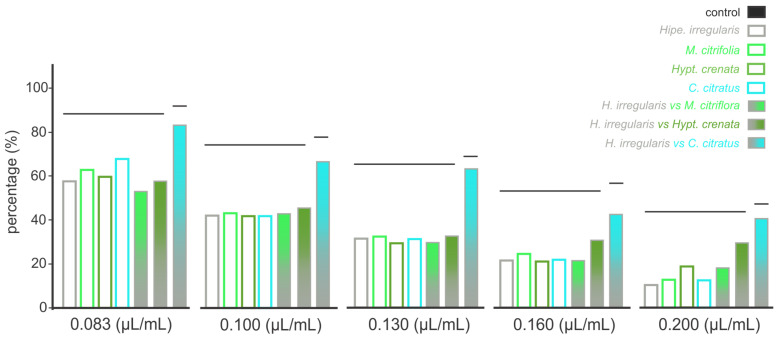
Oviposition deterrence in *Aedes aegypti* females mediated by the exposure to different concentrations of “alecrim do Cerrado” (*Hypenia irregularis*) pure essential oil and its combinations (1:1) with essential oils from noni (*Morinda citrifolia),* “salva-do-Marajó” (Brazilian mint; *Hyptis crenata*), and lemongrass (*Cymbopogon citratus*). The bars represent the percentage of laid eggs in arenas that received essential oil-containing solutions in comparison to the control (i.e., untreated solutions). The oviposition period was 14 days. Bars grouped at the same horizontal line indicate the absence of significant differences according to Tukey’s HSD test (*P* < 0.05).

The oviposition deterrent effect of the four essential oils was concentration dependent manner. At the lowest concentration (i.e., 0.083 μL/mL), oviposition was reduced by 50% compared to negative control (untreated solutions), while at the highest concentrations (i.e., 0.200 μL/mL), the reduction reached up to 80% (Figure 4, Supplementary Figure S2). Similar patterns were recorded for the mixtures (1:1 proportions) between the essential oils of *Hipe. irregularis* and *Hypt. crenata* or *M. citrifolia* (Figure 4, Supplementary Figure S2). The mixture between essential oils of *Hipe. irregularis* and *C. citratus* provided significant oviposition reductions only at concentrations above 0.130 μL/mL (Supplementary Figure S2), indicating potential antagonistic effects of the mixtures at the essential oil lowest concentrations (i.e., 0.083 μL/mL and 0.130 μL/mL) compared to the oviposition deterrence of the sole essential oils (Figure 4).

## 3. Discussion

Our results demonstrated that the essential oils of *Hype. irregularis*, *M. citrifolia*, *Hypt. crenata*, and *C. citratus* exhibit larvicidal activity against *A. aegypti* larvae, both individually and in 1:1 mixtures. In silico analyses indicated that 2,5-dimethoxy-*p*-cymene and carvacrol showed the strongest affinities for key *A. aegypti* targets, including AChE, OBP, and the odorant receptor AaOr31. No significant synergistic or antagonistic differences in LC_50_ values were recorded for essential oil mixture that combined *Hipe. irregularis* × *M. citrifolia*, *Hipe. irregularis* × *H. crenata*, and *Hipe. irregularis* × *C. citratus*, when compared to the larvicidal activities measured by the application of essential oils alone. When tested at 33.0 nL/cm^2^, all essential oils provided over 90% repellency against *A. aegypti* for up to 140 min, which was comparable to the repellence level achieved by the application of a commercial formulation containing DEET (15%). Similarly, no synergistic or antagonistic effects were reported in the repellence performance of essential oil mixtures that combined *Hipe. irregularis* × *M. citrifolia* and *Hipe. irregularis* × *Hypt. crenata*. However, the mixture that contained *Hipe. irregularis* × *C. citratus* essential oils resulted in reduced repellence and oviposition deterrence performances.

Chromatographic analysis revealed that the *Hipe. irregularis* × *M. citrifolia* mixture was rich in octanoic acid (23.0%), 2,5-dimethoxy-*p*-cymene (19.5%), and α-cymene (10%). The *Hipe. irregularis* × *Hypt. crenata* mixture was dominated by 2,5-dimethoxy-*p*-cymene (21.9%), carvacrol (10.4%), and α-cymene (10%). In the *Hipe. irregularis* × *C. citratus* mixture, the major compounds were citral A (23.0%), citral B (17.1%), and 2,5-dimethoxy-*p*-cimene (15.5%). Although thymol has been previously identified as the main compound (approximately 21%) in *Hipe. irregularis* essential oil [25], its concentration was drastically reduced (approximately 5%) in the mixture of *Hipe. irregularis* and *M. citrifolia* essential oils. Similarly, octanoic acid, normally the dominant (at least 64%) constituent [40,41] in *M. citrifolia* essential oils, remained prevalent in the mixture, but at reduced levels (approximately 23%). Likewise, 1,8-cineole and α-pinene, major components of *Hypt. crenata* [36,42], were absent in its mixture with *Hipe. irregularis*. Conversely, the chemical profile of *Hipe. irregularis* × *C. citratus* was consistent with known constituents of *C. citratus* essential oil [24,43]. These findings suggest that blending essential oils may alter their chemical composition due to interactions among constituents, thereby influencing biological activity. Although further investigations such as in-depth comparisons of the chemical composition of the mixed and individual essential oils, the chemotypes of individual plant populations, and electrophysiological and ligand-binding assays, are still needed to draw definitive conclusions, the reduced levels of thymol, α-cymene, and octanoic acid in the mixture of *Hype. irregularis* and *M. citrifolia* essential oils may help explain the antagonistic effects observed on repellence and oviposition deterrence of this essential oil mixture at the lowest concentrations. These compounds are typically present at higher concentrations in the pure essential oils.

The toxicity observed in the *Hipe. irregularis* × *M. citrifolia* mixture is likely attributable to its major compounds. Octanoic acid has been reported to have insecticidal [44], bactericidal [45], nematicidal [46], and fungicidal [40,41,47] properties and may act synergistically with other compounds [48]. Likewise, essential oils containing 2,5-dimethoxy-*p*-cymene have demonstrated insecticidal [49,50] and bactericidal [51,52] activity. The larvicidal effect of the *Hipe. irregularis* × *Hypt. crenata* mixture may be due not only to 2,5-dimethoxy-*p*-cymene but also to carvacrol and α-cymene. Carvacrol has broad bioactivity, including bactericidal, acaricidal, and insecticidal effects, and is known to be toxic to disease vectors [53,54,55]. Although our in silico results suggest only moderate affinity of carvacrol for AChE, other studies have shown it to modulate TRP channels, inhibit AChE, act as a positive allosteric modulator of GABA receptors [56,57], and noncompetitively block nicotine binding to nAChRs, leading to neuroinhibition [58,59,60]. It has also been proposed that carvacrol can block the octopamine receptor pathway [61]. Citral, predominant in the *Hipe. irregularis* × *C. citratus* mixture, has been shown to inhibit AChE and β-esterase activity in insects [58,62,63].

All essential oils tested exhibited over 90% repellency against *A. aegypti* when applied at 0.0330 µL/cm^3^, maintaining effectiveness for 140 min—comparable to DEET. The repellent activity of *H. irregularis*, *M. citrifolia*, *H. crenata*, and *C. citratus* has been previously documented against various insect species [25,26,27,32,64], but studies on their combined repellent effects are lacking. The repellency observed in the *Hipe. irregularis* × *M. citrifolia* mixture may be due to octanoic acid and 2,5-dimethoxy-*p*-cymene, both associated with arthropod repellency [30,34]. Carvacrol- and α-cymene-containing oils have also shown repellent activity, with carvacrol alone proven effective against mosquitoes and other arthropods [6,31,65,66,67]. Citral is a well-known insect repellent [24,32,65,68], and its efficacy has been demonstrated in pure form [69,70]. Indeed molecular docking analyses revealed that 2,5-dimethoxy-*p*-cymene and carvacrol interacted with OBP and the odorant receptor AaOr31, which are involved in insect chemoreception and play key roles in olfaction [71,72]. Thireou et al. [73] and Kröber et al. [74] reported strong carvacrol binding to OBPs in *Anopheles gambiae*, including the same OBP that interacts with DEET and Icaridin. Carvacrol also binds to AgamOBP5 with high affinity [75,76]. AaOr31 has been shown to respond to β-farnesene and mediate pyrethrum repellency in *A. aegypti* [77,78]. Therefore, these receptors may serve as targets for terpene-based repellents such as 2,5-dimethoxy-*p*-cymene and carvacrol. Interestingly, Lü and Liu [33] reported that citral can be attractive at low concentrations but repellent at higher doses. While no synergistic or antagonistic repellent effects were seen in *Hipe. irregularis* × *M. citrifolia* or *Hipe. irregularis* × *Hypt. crenata* combinations, reduced repellency was observed in the *Hipe. irregularis* × *C. citratus* blend. Although some studies have demonstrated synergistic effects of *C. citratus* [79,80], others have reported antagonistic interactions when it is combined with other essential oils [81]. These outcomes likely depend on the specific chemical makeup of the oils, their component interactions, and the target organisms.

A last note is that all tested essential oils also showed concentration-dependent oviposition deterrent activity. But no synergistic or antagonistic effects were noted, except for the *Hipe. irregularis* × *C. citratus* combination, which led to increased oviposition—indicating a possible antagonistic effect. Fatty acids, especially octanoic acid, have previously been linked to oviposition deterrence [82,83,84], although some studies have reported the opposite effect [85,86], highlighting species-specific and context-dependent responses. Similarly, essential oils containing carvacrol, as well as carvacrol alone, have demonstrated oviposition deterrence similar to octanoic acid [87,88,89]. Comparable results have also been observed for citral [63,90]. Overall, the predicted differences in binding affinities between the major compounds of the essential oil mixtures and various neurophysiological targets support the hypothesis of multiple modes of action, a beneficial feature for developing environmentally safe biorational insecticides. These findings should be viewed as exploratory and hypothesis-generating.

## 4. Conclusions

Our findings highlight the potential of essential oils from *H. irregularis*, *M. citrifolia*, *H. crenata*, and *C. citratus*, all of which are widely available in Brazil, as sustainable sources of biorational products for controlling *A. aegypti*. These oils exhibit promising dual functionality: acting as larvicidal agents and serving as preventive tools through their oviposition deterrent and repellent properties. The predicted binding affinities suggest multiple modes of action, a beneficial trait for developing environmentally safe biorational insecticides. Although exploratory, our findings provide a basis for future validation through electrophysiological or biochemical assays. Additional targets, such as voltage-gated sodium channels, ryanodine receptors, and nicotinic acetylcholine receptors, may also be involved. Collectively, the findings described here offer valuable insights into the potential mechanisms of essential oil-based insecticides and support their continued investigation.

## 5. Materials and Methods

### 5.1. Essential Oil Extraction

Branches of *Hipe. irregularis*, native to the Central Brazilian Cerrado, containing leaves and flowers were collected in Jalapão, Tocantins (09°57′46″ S, 47°40′38″ W), a region characterized by nutrient-poor, acidic soils and a strongly seasonal climate, which influence the species’ secondary metabolism. In contrast, the other plant species (i.e., *C. citratus*, *Hypt. crenata*, and *M. citrifolia*) were collected at the Federal University of Tocantins, campus of Gurupi (11°43′45″ S, 49°04′07″ W), where edaphoclimatic conditions differ considerably, with generally more fertile soils and a more humid tropical climate. Essential oils were extracted separately from each plant species using healthy leaves that were shade-dried for a period of 10 days. The dried leaves of each species were then cut into small pieces. For each extraction, 200 g of dried leaves were combined with 800 mL of distilled water in a 1000 mL round-bottomed flask and subjected to hydrodistillation using a Clevenger apparatus for three hours. The essential oils were collected individually in amber bottles and stored at 4 °C [91].

### 5.2. Gas Chromatography (GC) Analysis

The chemical composition of the essential oils was determined at the Analytical Center of the Chemistry Institute, University of São Paulo, using GC–MS. Analyses were performed on a Shimadzu GC-2010 instrument equipped with a QP2010Plus mass selective detector. The GC was fitted with a fused silica capillary column (30 m × 0.25 mm × 0.25 μm film thickness), with the following temperature program: 60 to 240 °C at 3 °C/min. Injector temperature was set to 220 °C. Helium was used as the carrier gas, and the injection was performed in split mode (1:100), using 1 µL of a 1:1000 solution in hexane. For the MS, the following parameters were used: electron impact ionization at 70 eV, with the ion source and interface temperatures set at 200 °C [92]. The components of the essential oils were identified by comparing their mass spectra with those in the spectrophotometer database (Wiley 7, NIST 05, and NIST 05s) and by analyzing their retention indices (RI). To calculate the RI, a mixture of saturated C7–C40 alkanes (Supelco Inc., Bellefonte, PA, USA) was analyzed under the same chromatographic conditions as the essential oil, and the adjusted retention times of the compounds were determined. The resulting RI values were then compared with those reported in the literature [93,94].

### 5.3. Origin and Maintenance of Aedes aegypti Mosquitoes

*Aedes aegypti* mosquitoes as used in this study were obtained from local populations collected in Gurupi City (11°43′07″ S, Tocantins State, Brazil) and maintained for several generations at the Laboratory of Integrated Pest Management, Federal University of Tocantins, Campus Gurupi. Adult males were fed a 10% sucrose solution, while adult females were fed on heparinized horse blood. Larvae were reared in plastic containers (35 cm × 5 cm) and fed a sterilized diet consisting of an 80:20 mixture of chick chow and yeast [95].

### 5.4. Bioassays of Toxicity

Toxicity tests were conducted following the World Health Organization protocol [96], with minor modifications. A stock solution was prepared at a concentration of 10 µL/mL using 1.7% dimethyl sulfoxide (DMSO) in distilled water. From this stock, a series of working concentrations was prepared (0.007, 0.013, 0.020, 0.027, 0.030, 0.050, 0.067, 0.083, 0.100, and 0.130 µL/mL). For each concentration, 30 mL of the test solution and 25 third-instar *A. aegypti* larvae were placed into disposable 100 mL plastic cups. Three replicates were performed per concentration.

The bioassays were maintained at 27 ± 1 °C, 65 ± 6% relative humidity, with a 12 h photoperiod. After 24 h, larval mortality was recorded, and the median lethal concentration (LC_50_) was estimated. Using the same procedure, we evaluated potential synergistic effects between *Hipe. irregularis* and *M. citrifolia*, *H. crenata*, or *C. citratus* in terms of toxicity, as outlined in Table 5.

### 5.5. Molecular Modeling Analysis

The ligands selected for the molecular docking study were the major compounds found in combinations of *Hipe. irregularis* with *M. citrifolia*, *H. crenata*, and *C. citratus*. The 3D structures of these compounds, in their neutral forms, were constructed using Marvin Sketch 18.10 (ChemAxon, http://www.chemaxon.com).

Amino acid sequences of the target proteins were obtained from the National Center for Biotechnology Information (NCBI) database (https://www.ncbi.nlm.nih.gov/, accessed on 30 October 2024). Their 3D structures were constructed via homology modeling using the Swiss-Model Workspace (https://swissmodel.expasy.org/, https://www.ncbi.nlm.nih.gov/, accessed on 30 October 2024), following the selection of appropriate templates with the BLASTp tool. Templates were obtained from the Protein Data Bank (https://www.rcsb.org/, https://www.ncbi.nlm.nih.gov/, accessed on 30 October 2024), considering quality parameters such as experimental method, resolution, R-value, and complexation with a ligand. The Swiss-Model platform was also used to verify structural integrity and active site conformation [97]. Model validation was performed using Ramachandran plots [98,99] to assess the distribution of backbone torsion angles (ϕ and ψ), thereby evaluating stereochemical quality. The QMEAN score was also used to assess model reliability [100].

Protein targets and ligands were prepared for docking using AutoDock Tools 1.5.7 [101], following the methodology proposed by Souza Moura et al. [102]. Docking calculations were performed using AutoDock Vina [103], generating nine binding poses for each ligand–target interaction and reporting the binding affinity in kcal/mol. The best docking poses were selected based on binding affinity and were visualized and analyzed using PyMOL 2.0 [104] and Discovery Studio 4.5 [105].

### 5.6. Repellence Test

The repellency test was conducted using the essential oils of *Hype irregularis*, *M. citrifolia*, *Hypt. crenata*, and *C. citratus*, following the method described by Haris, Azeem, and Binyameen [4]. Three acrylic boxes (24 × 24 × 24 cm) were used: one for testing the essential oils, one as a negative control (ethanol), and one as a positive control (15% DEET). Each box contained 50 adult female *A. aegypti* mosquitoes aged 5–7 days. In parallel, forearms of five volunteers were cleaned with neutral soap, sanitized with 70% ethanol, and dried. A 300 cm^2^ area on each forearm was marked for treatment and exposure; the remaining areas were covered with rubber gloves. Essential oil solutions were prepared at concentrations of 0.0033, 0.017, 0.033, 0.067, 0.167, 0.333, and 0.500 μL/cm^2^ using 99.80% ethanol at a 1:1 ratio. These were applied to the exposed skin areas. Finally, forearms were inserted into the acrylic boxes for 3 min every 30 min, and the number of mosquito bites was recorded. After 140 min, the total number of bites was used to estimate protective efficacy. The same procedure was used to evaluate the synergistic effects of oil combinations (1:1 ratio). Five replicates were performed per concentration, and all tests were conducted during daytime hours. The repellence index was calculated using the formula:

%RI = ((T − I)/T) × 100(1)

where RI is the percentage of repellence, T is the number of bites in the control, and I is the number in the treatment (essential oil protection).

We tested each essential oil alone and the mixture formulations on human volunteer subjects, following approval of the research protocol number CAAE 81727617.3.0000.0003 (https://plataformabrasil.saude.gov.br/, accessed on 4 August 2025, approved on 23 March 2018).

### 5.7. Oviposition Deterrence Test

Oviposition deterrence was evaluated using essential oil concentrations of 0.0833, 0.1, 0.13, 0.166, and 0.2 μL/mL, prepared as previously described. Synergistic effects of essential oil combinations (1:1 ratio) were also assessed at the same concentrations. For the experiment, entomological cages (35 cm wide × 23 cm deep × 47 cm high) were used. Each cage contained two disposable cups: one with 30 mL of the essential oil solution (pure or in combination), and the other with distilled water containing DMSO as the control. Cups were wrapped in aluminum foil to prevent visual bias. Twenty-five newly emerged female and fifty male *A. aegypti* mosquitoes were released into each cage. Mosquitoes were fed daily with rodent blood and a 10% sucrose solution and maintained at 28 °C. Egg counts were recorded daily over seven days. The same procedure was followed to assess the effects of oil combinations. Oviposition deterrence was calculated using the formula:

%V = ((T − I)/T) × 100(2)

where V is the percentage of oviposition deterrence, T is the number of viable eggs in the control, and I is the number in the treatment.

### 5.8. Statistical Analysis

Lethal concentrations (LC_50_ and LC_95_) were estimated using the PROBIT analysis method with POLO PLUS statistical software (version 1.0, LeOra Software, Berkeley, CA, USA). By means of SigmaPlot 14.0 software (Systat Software, San Jose, CA, USA), we applied analysis of variance (ANOVA) and Tukey’s HSD test (*p* < 0.05) to compare results between treatment groups in the repellence and oviposition deterrence analysis using SigmaPlot 14.0 (Systat Software, San Jose, CA, USA).

## Figures and Tables

**Table 1:** Relative percentage (area%), obtained by gas chromatography coupled to mass spectrometry (GC–MS) detector of the constituents of the essential oils from the dried leaves of *Hypenia irregularis* x *Morinda citrifolia*, *Hyptis crenata* and *Cymbopogon citratus*.

Constituents	*Hypenia irregularis* x *Morinda Citrifolia*			*Hypenia irregularis* x *Hyptis Crenata*			*Hypenia irregularis* x *Cymbopogon Citratus*		
	TR	IR	(%)	TR	IR	(%)	TR	IR	(%)
Diacetone alcohol	2.91	2.90	9.45	2.93	2.90	9.42	2.92	2.88	9.34
Hexanoic acid	4.64	4.55	2.45	-	-	-	-	-	-
β-myrcene	4.85	4.82	1.00	4.85	4.80	1.76	4.85	4.80	4.11
α-cymene	5.41	5.33	10.0	4.85	4.80	10.0	5.41	5.35	7.34
α-pinene	-	-	-	4.12	4.01	1.47	-	-	-
α-terpineol	-	-	-	7.97	7.95	0.93	-	-	-
α-bergamotene	-	-	-	-	-	-	11.2	11.2	0.30
Eucalyptol	-	-	-	5.54	5.50	4.77	-	-	-
Terpinene	-	-	-	5.91	5.87	1.1	-	-	-
Camphor	-	-	-	7.3	7.26	9.21	-	-	-
Linalool	6.50	6.46	0.80	6.5	6.47	1.12	6.50	6.46	0.97
Sulcatone	-	-	-	-	-	-	4.77	4.73	0.26
Isogeraniol	-	-	-	-	-	-	7.69	7.64	0.41
Citral B, neral	-	-	-	-	-	-	8.56	8.51	17.1
Geraniol	-	-	-	-	-	-	8.70	8.61	1.44
Citral A, geranial	-	-	-	-	-	-	8.97	8.93	23.0
Octanoic acid, methyl ester	6.83	6.80	0.79	-	-	-	-	-	-
Octanoic acid	7.64	7.40	23.0	-	-	-	-	-	-
Terpinene-4-ol	7.77	7.73	0.70	7.78	7.78	1.65	7.77	7.74	0.44
Benzene, 2-methoxy-4-methyl-1-(1-methylethyl)	8.43	8.40	2.50	-	-	-	-	-	-
Anisol	8.60	8.53	1.94	8.43	8.40	2.9	8.43	8.40	1.98
Hexanoic acid, 4-pentenyl ester	8.83	8.80	2.07	-	-	-	-	-	-
Thymol	9.27	9.23	4.30	-	-	-	-	-	-
Carvacrol	-	-	-	9.4	9.35	10.4	9.39	9.35	6.87
2,5-dimethoxy-*p*-cimene	10.9	10.9	19.5	10.9	10.9	21.9	10.9	10.9	15.5
Caryophyllene	11.2	11.1	1.21	11.1	11.1	2.58	11.1	11.1	0.84
Isobutyl-3-methylbut-3-enyl carbonate	11.5	11.5	3.05	-	-	-	-	-	-
Humulene	11.6	11.5	3.02	11.6	11.5	3.52	11.6	11.5	2.13
Phenol, 3-(1,1-dimethylethyl)-4-methoxy	11.8	11.8	3.94	13.2	13.1	1.05	13.3	13.1	0.45
**(1R,3E,7E,11R)-1,5,5,8-Tetramethyl-12-oxabicato**	13.5	13.5	0.41	**-**	**-**	**-**	**-**	**-**	**-**
3-tert-butyl-4-hydroxyanisole	**-**	**-**	**-**	11.8	11.7	1.49	11.7	11.7	0.91
**Monoterpenes**	**-**	**-**	**18.56**	**-**	**-**	**42.90**	**-**	**-**	**56.69**
**Sesquiterpenes**	**-**	**-**	**6.47**	**-**	**-**	**6.10**	**-**	**-**	**3.27**
**Phenylpropanoids**	**-**	**-**	**36.23**	**-**	**-**	**38.69**	**-**	**-**	**24.75**
**Others**	**-**	**-**	**38.71**	**-**	**-**	**9.90**	**-**	**-**	**15.22**
**Total**			**99.97**			**97.59**			**99.93**

TR: time retention; IR: Index Retention.

**Table 2:** Toxicity of essential oils of Hypenia irregularis, Morinda citrifolia, Hyptis crenata, and Cymbopogon citratus against larvae of Aedes aegypti.

Essential Oil	Slope ± SE	LC_50_ (CI 95%) (μL/mL)	LC_95_ (CI 95%) (μL/mL)	*χ* ^2^	*P*
*Hypenia irregularis*	2.53 ± 0.280	0.037 (0.020–0.048)	0.122 (0.079–0.221)	8.203	0.64
*Morinda citrifolia*	2.71 ± 0.292	0.036 (0.019–0.049)	0.120 (0.079–0.217)	9.223	0.51
*Hyptis crenata*	2.37 ± 0.273	0.040 (0.029–0.045)	0.126 (0.081–0.234)	8.968	0.67
*Cymbopogon citratus*	1.93 ± 0.257	0.051 (0.048–0.079)	0.137 (0.113–0.301)	8.272	0.28

SE: Standard error; LC_50_: Lethal concentrations capable of killing 50% of tested larvae; LC_95_: Lethal concentrations capable of killing 95% of tested larvae; CI 95%: confidence interval.

**Table 3:** Estimated lethal concentrations (LC) of essential oil mixtures containing *Hypenia irregularis* x *Morinda citrifolia*, *Hipe. irregularis* x *Hyptis crenata*, and *Hipe. irregularis* x *Cymbopogon citratus* against larvae of *Aedes aegypti*.

	Proportions	Slope ± SE	LC_50_ (CI 95%) (μL/mL)	LC_95_ (CI 95%) (μL/mL)	*χ^2^*	*P*
*Hipe. irregulares* × *M. citriflora*	0:1	2.71 ± 0.292	0.036 (0.019–0.049)	0.120 (0.079–0.217)	9.23	0.51
	1:2	2.08 ± 0.240	0.063 (0.046–0.066)	0.147 (0.106–0.244)	8.64	0.62
	1:3	2.49 ± 0.292	0.049 (0.032–0.057)	0.139 (0.098–0.236)	8.35	0.66
	1:1	2.46 ± 0.277	0.032 (0.015–0.041)	0.118 (0.077–0.215)	9.00	0.55
	1:0	2.53 ± 0.280	0.037 (0.020–0.048)	0.122 (0.081–0.219)	8.20	0.64
	2:1	2.05 ± 0.301	0.061 (0.044–0.069)	0.144 (0.079–0.221)	8.53	0.81
	3:1	2.17 ± 0.118	0.054 (0.037–0.072)	0.142 (0.101–0.239)	8.94	0.74
*Hipe. irregulares* × *Hypt. crenata*	0:1	2.37 ± 0.273	0.040 (0.029–0.045)	0.126 (0.081–0.234)	8.27	0.67
	1:2	2.81 ± 0.341	0.068 (0.052–0.071)	0.161 (0.116–0.305)	8.33	0.50
	1:3	2.74 ± 0.233	0.055 (0.039–0.067)	0.147 (0.102–0.291)	8.91	0.63
	1:1	2.63 ± 0.180	0.037 (0.021–0.052)	0.124 (0.079–0.268)	8.22	0.59
	1:0	2.53 ± 0.280	0.037 (0.020–0.048)	0.122 (0.079–0.221)	8.21	0.64
	2:1	2.35 ± 0.203	0.065 (0.049–0.069)	0.153 (0.108–0.297)	8.72	0.88
	3:1	2.24 ± 0.103	0.049 (0.033–0.055)	0.141 (0.096–0.285)	8.99	0.94
*Hipe. irregularis* × *C. citratus*	0:1	1.93 ± 0.257	0.051 (0.045–0.079)	0.143 (0.113–0.301)	8.27	0.28
	1:2	1.53 ± 0.393	0.074 (0.065–0.093)	0.169 (0.139–0.327)	8.47	0.49
	1:3	1.53 ± 0.123	0.059 (0.053–0.081)	0.148 (0.118–0.306)	8.83	0.31
	1:1	2.00 ± 0.280	0.049 (0.039–0.067)	0.139 (0.109–0.297)	8.30	0.44
	1:0	2.53 ± 0.201	0.034 (0.020–0.048)	0.118 (0.079–0.221)	8.21	0.64
	2:1	2.18 ± 0.332	0.071 (0.063–0.091)	0.162 (0.132–0.320)	9.12	0.51
	3:1	2.21 ± 0.238	0.050 (0.041–0.069)	0.140 (0.110–0.298)	9.01	0.39

SE: Standard error; LC_50_: Lethal concentrations capable of killing 50% of tested larvae; LC_95_: Lethal concentrations capable of killing 95% of tested larvae; CI 95%: confidence interval.

**Table 4:** Molecular docking results for complexes between major compounds and target of *Aedes aegypti*.

Essential Oil	Ligand	Affinity Energy (kcal/mol)					
		AChE	AeagOBP1	AaOR31	GABAR	TRP	OCT
*Hipenia irregularis* x *Morinda citrifolia*	Octanoic acid	−4.9	−5.8	−4.8	−3.9	−4.1	−4.2
	2,5-dimethoxy-*p*-cymene	−6.6	−6.8	−6.5	−4.5	−5.4	−5.2
*Hipenia irregularis* x *Hyptis crenata*	2,5-dimethoxy-*p*-cymene	−6.6	−6.8	−6.5	−4.5	−5.4	−5.2
	Carvacrol	−6.1	−7.0	−6.4	−5.0	−5.2	−5.6
*Hipenia irregularis* x *Cymbopogon citratus*	Citral	−5.5	−5.7	−5.3	−4.1	−4.3	−4.5
	2,5-dimethoxy-*p*-cymene	−6.6	−6.8	−6.5	−4.5	−5.4	−5.2

AChE: acetylcholinesterases; AeagOBP1: odorant-binding protein of *Aedes aegypti*; AaOR31: olfactory receptor 31 of *Aedes aegypti*; GABAR: receptors for γ-aminobutyric acid; TRP: transitory receptor potential channels; OCT: receptor for octopamine.

**Table 5:** Proportions tested to determine the synergistic/antagonistic effects of *Hypenia irregularis* with *Morinda citrifolia*, *Hyptis crenata*, and *Cymbopogon citratus*.

Proportions	*Hipe. Irregularis* (µL/mL)	Essential Oil x, y, z (µL/mL)
0:1	0	100
1:1	50	50
2:1	67	33
3:1	75	25
1:0	100	0
1:2	33	67
1:3	25	75

x: Morinda citrifolia, y: Hyptis crenata, z: Cymbopogon citratus.

## Data Availability

The original contributions presented in this study are included in the article/Supplementary Material. Further inquiries can be directed to the corresponding authors.

## Supplementary Materials

The following supporting information can be downloaded at: https://www.mdpi.com/article/10.3390/toxins17080402/s1, Figure S1: Carvacrol, citral, 2,5-dimethoxy-*p*-cymene, and octanoic acid bind with the GABA receptor (**A**), TRP channel (**B**) and Octopamine receptor (**C**) target complexes of *Aedes aegypti*; the 2D maps of molecular interactions with amino acids in each target active site (yellow) are also shown; Figure S2: Oviposition deterrence in *Aedes aegypti* females mediated by the exposure to different concentrations of “alecrim do Cerrado”, *Hypenia irregularis*, essential oil and its combinations (1:1) with essential oils from noni (*Morinda citrifolia*), (“salva-do-Marajó (Brazilian mint; *Hyptis crenata*), and lemongrass (*Cymbopogon citratus*). The bars represent the number of laid eggs in arenas that received essential oil-containing solutions in comparison to the control (i.e., untreated solutions). The oviposition period was 14 days; Table S1: Phytochemical profiles for essential oils of *Hypenia irregularis*, *Morinda citrifolia*, *Hyptis crenata* and *Cymbopogon citratus* revealed by gas chromatography coupled to mass spectrometry (GC-MS) detectors. These results were obtained in publications available in the literature; Table S2: Target models of *Aedes aegypti* used to analyze the molecular docking with the major compounds. Refs. [25,40] are also cited in the Supplementary Materials. Refs. [106,107] are cited only in the Supplementary Materials.

## Abbreviations

Dimethyl sulfoxide	DMSO
N,N-diethyl-meta-toluamide	DEET
*Hypenia iregularis*	*Hypt. irregularis*
*Morinda citrifolia*	*M. citrifolia*
*Hyptis crenata*	*Hypt. crenata*
*Cymbopogom citratus*	*C. citratus*
*Aedes aegypti*	*A. aegypti*
Acetylcholinesterase	AChE
Glutathione S-transferase	GST
γ-aminobutyric acid	GABA
Transient receptor potential	TRP
Gas Chromatography coupled with Mass Spectrometry	GC–MS
