# Supplementary material for: Phytochemistry, Mode of Action Predictions, and Synergistic Potential of Hypenia irregularis Essential Oil Mixtures for Controlling Aedes aegypti
Source: Toxins (Basel). 2025 Aug 11;17(8):402. doi: 10.3390/toxins17080402 (PMC12390512; doi:10.3390/toxins17080402)
Supplement: Supplementary file 1 [file toxins-17-00402-s001.zip › toxins-3741966-supplementary.pdf]

toxins

MDPI

Supplementary Materials: Phytochemistry, Mode of Action Predictions, and Synergistic Potential of *Hypenia irregularis* Essential Oil Mixtures for Controlling *Aedes aegypti*

Luis O. Viteri, Wellington S. Moura, Richard D. Possel, Osmany M. Herrera, Rodrigo R. Fidelis, Bruno S. Andrade, Guy Smagghe, Gil R. Santos, Eugenio E. Oliveira, Raimundo W. S. Aguiar.

Molecular interaction descriptions

The interactions of the tested compounds with the GABA receptor, TRP channel, and octopamine receptor revealed distinct molecular binding characteristics (Supplementary Figure S1). In the GABA receptor, carvacrol binds predominantly through pi-alkyl and van der Waals interactions with VAL143, PHE201, GLU145, and HIS142, indicating a largely hydrophobic interaction profile. Citral engages in conventional hydrogen bonds and pi-alkyl interactions with residues such as SER200, TYR249, ARG95, and THR248, suggesting a more polar binding pattern. 2,5-dimethoxy-*p*-cymene displays van der Waals and pi-alkyl interactions with PHE141, GLU145, and PHE201, supporting a stable hydrophobic binding configuration. Octanoic acid interacts mainly through hydrogen bonds with THR137, TYR149, and SER91, highlighting a more hydrophilic interaction network involving polar residues. Regarding the TRP channel, carvacrol exhibits pi-pi T-shaped, pi-alkyl, and van der Waals interactions with ALA301, HIS250, ASN320, and TRP299, forming a well-anchored configuration within the binding site. Citral establishes alkyl and pi-sigma interactions with LEU703, ARG705, ARG707, and GLU709, indicating moderate affinity and flexible orientation. 2,5-dimethoxy-*p*-cymene interacts via van der Waals and hydrophobic contacts with GLY564, VAL561, PHE702, and TRP480, suggesting a favorable binding conformation. Octanoic acid binds through van der Waals forces and pi-alkyl interactions involving HIS548, GLY560, ARG565, and ALA703, confirming a compatible fit within the hydrophobic environment of the channel. In the octopamine receptor, carvacrol forms pi-alkyl and van der Waals interactions with ILE65, VAL216, VAL257, and PRO261, establishing a stable hydrophobic engagement. Citral engages through a combination of alkyl, pi-alkyl, and conventional hydrogen bonding with THR629, ILE300, LEU633, and TYR634, indicating a broad and diverse interaction network. 2,5-dimethoxy-*p*-cymene interacts with VAL218, ILE217, GLY256, and LEU62 via van der Waals and pi-alkyl contacts, reflecting a favorable hydrophobic binding profile. Octanoic acid binds through both hydrogen bonding and alkyl interactions, particularly with ARG303, TYR269, ASN270, and PHE245, confirming its compatibility with both polar and nonpolar regions of the receptor.

 https://doi.org/10.3390/toxins17080402

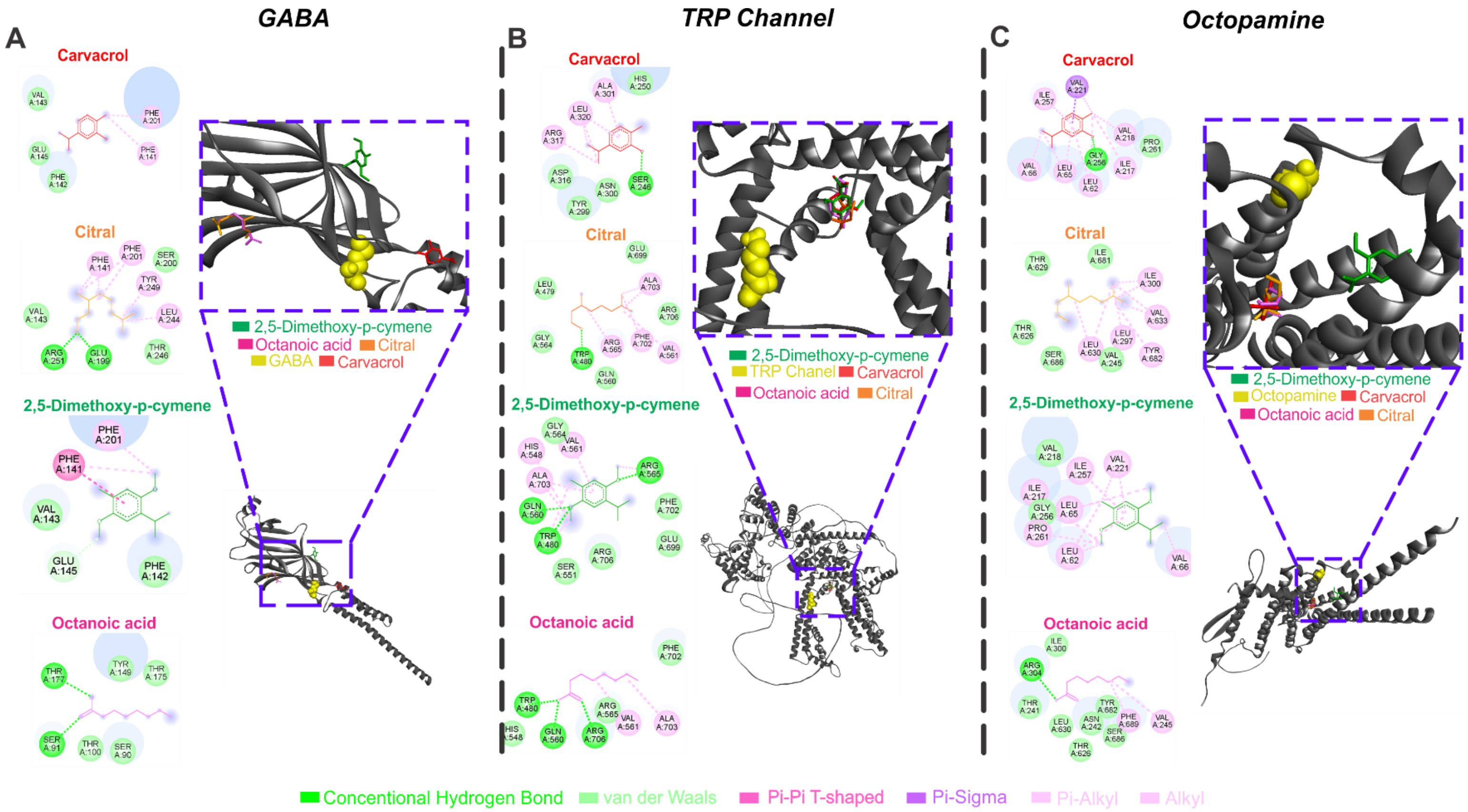

Figure S1. Carvacrol, citral, 2,5-dimethoxy-p-cymene, and octanoic acid bind with the GABA receptor (A), TRP channel (B) and Octopamine receptor (C) target complexes of *Aedes aegypti*; the 2D maps of molecular interactions with amino acids in each target active site (yellow) are also shown.

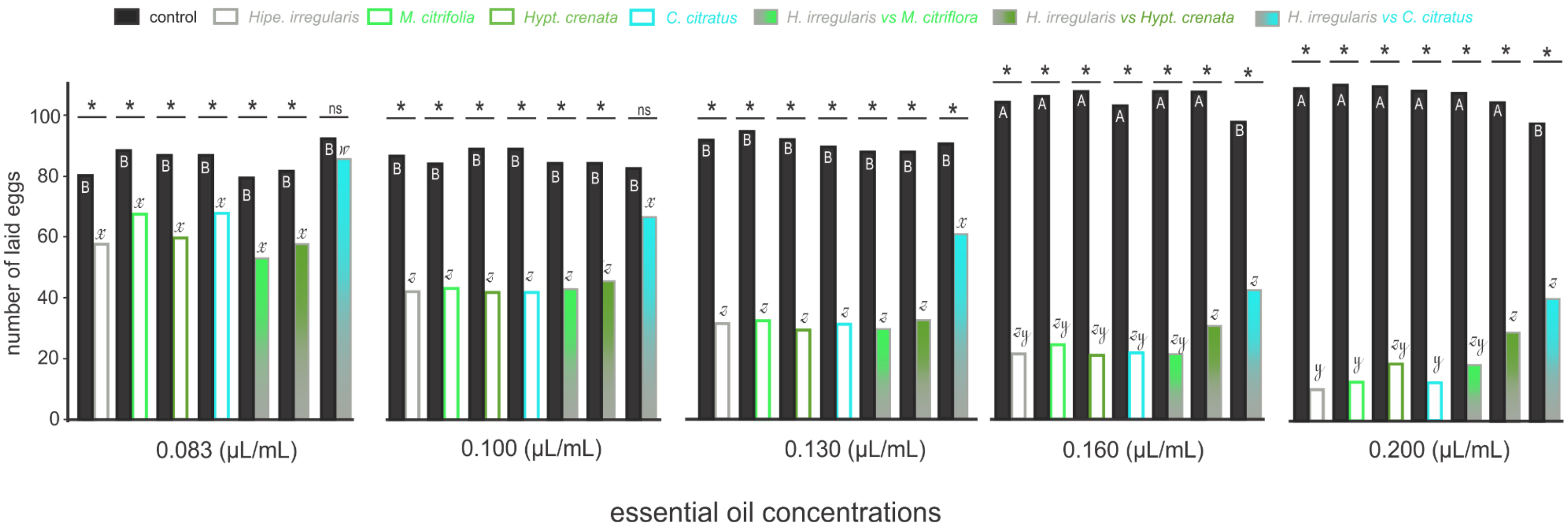

Figure S2. Oviposition deterrence in *Aedes aegypti* females mediated by the exposure to different concentrations of "alecrim do Cerrado (*Hypenia irregularis*), pure essential oil and its combinations (1:1) with essential oils from noni (*Morinda citrifolia*), ("salva-do-Marajó (Brazilian mint; *Hyptis crenata*), and lemongrass (*Cymbopogon citratus*). The bars represent the number of laid eggs in arenas that received essential oil-containing solutions in comparison to the control (i.e., untreated solutions). The oviposition period was 14 days. Bars grouped with the same capital letter indicate the absence of significant differences according to Tukey's HSD test ($P < 0.05$). Similarly, bars grouped with the same symbol letter indicate the absence of significant differences according to Tukey's HSD test ($P < 0.05$). Asterisks means significant difference in paired *t*-test ($P < 0.05$). *ns*: non-significant difference (paired *t*-test $P < 0.05$).

Table S1. Phytochemical profiles for essential oils of *Hypenia irregularis*, *Morinda citrifolia*, *Hyptis crenata* and *Cymbopogon citratus* revealed by gas chromatography coupled to mass spectrometry (GC-MS) detectors. These results were obtained in publications available in the literature.

	***Hypenia irregularis* [1]**			***Morinda citriflora* [2]**			***Hyptis crenata* [3]**			***Cymbopogon citratus* [4]**		
**Constituents**	**TR**	**IR**	**(%)**	**TR**	**IR**	**(%)**	**TR**	**IR**	**(%)**	**TR**	**IR**	**(%)**
Diacetone alcohol	2.93	2.90	4.91									
Bicyclo [3.1.0] hex-2-eno, 2-methyl-5-(1-methylethyl)	4.01	3.98	0.64									
α-pinene	4.13	4.10	0.57									
Myrcene	4.87	4.83	2.04									
o-Cymene	5.43	5.38	15.6									
Linalool	6.51	6.47	1.43									
Bicyclo [3.1.0] hexan-3-one, 4-methyl-1-(1-methylethyl)	7.60	7.56	0.65									
Terpinen-4-ol	7.78	7.75	0.98									
Benzene, 2-metoxi-4-metil-1-(1-metiletil)	8.44	8.40	4.20									
Benzene, 2-methoxy-1-methyl-4- (1-methylethyl)	8.58	8.53	3.32									
Thymol	9.28	9.23	7.11									
Copaene	10.6	10.5	0.48									
2,5-dimethoxy-p-cymene	10.9	10.9	27.0									
Caryophyllene	11.2	11.1	2.08									
Bicyclo [3.1.1] hept-2-eno, 2,6-dimethyl-6-(4-methyl)	11.3	11.2	0.49									
Humulene	11.7	11.6	5.01									
Phenol, 3- (1,1-dimethylethyl) -4-methoxy	11.8	11.7	8.90									
1,5,5,8-tetramethyl-12-oxabicide	13.5	13.5	0.41									
2-hexanone,5-methyl-				3.55	3.66	0.22						
Hexanoic acid, methyl ester				3.9	4.12	1.27						
Hexanoic acid				5.11	5.55	12.7						

Benzene, tert-butyl-	5.45	5.54	0.08		
Butanoic acid,4-pentenyl ester	6.01	6.12	0.19		
2-Hexanone, 5-methyl-	6.41	6.50	0.03		
(E)-2-Methylbut-2-en-1-yl isobutyrate	6.53	6.63	0.05		
Octanoic acid, methyl ester	6.36	7.05	2.91		
Cyclopropanes,1,2,3-trimethyl-	7.33	7.40	0.07		
Octanoic acid	8.13	8.64	75.7		
Citronellol	8.44	8.50	0.03		
Hexanoic acid, 4-pentenyl ester	8.86	9.00	2.57		
1-Pentene, 5-(pentyloxy)-	9.31	9.39	0.37		
Pentane, 2,2′-oxybis-	9.42	9.46	0.05		
Decanoic acid, methyl ester	9.71	9.78	0.15		
Hexanoic acid, hexyl ester	10.55	10.61	0.07		
Isobutyl pent-4-enyl carbonate	11.49	11.82	3.12		
Dodecanoic acid, 2-penten-1-yl ester	11.97	12.20	0.31		
α-pinene				12.38	15.24
Camphene				13.21	3.23
ϱ-cymene				17.46	6.85
l-Limonene				17.72	3.94
1.8-cineole (Eucalyptol)				17.91	19.76
γ-terpinene				19.51	1.73
Camphor				24.89	33.62
β-caryophyllene				40.91	8.00
Aromadendrene				42.07	2.95
Ledene				45.65	0.99
Caryophyllene oxide				47.91	0.84
Viridiflorol				48.05	0.85
10-epi-γ-eudesmole				48.45	0.96

Caryophyllene oxide	48.87		1.05			
Mircene				7.74	986	9.73
(Z)-β-oxime				9.34	1020	0.32
(E)-β-oxime				9.75	1029	0.16
Linalool				11.84	107	1.64
Neral				17.88	1209	32.43
Geraniol				18.37	1220	4.52
Geranial				19.23	1239	41.46
2-undecanone				20.09	1359	0.35
Geranyl acetate				23.73	1443	0.42
E-Caryofylene				31.99	1641	0.17
others				-	-	8.80

TR: time retention; IR: Index Retention.

Table S2. Target model of *Aedes aegypti* used to analyze the molecular docking with the major compounds.

Receptor (NCBI database)	Model		Identity (%)	Ramachandran favored (%)	QMEAN
Acetylcholinesterase	XP_021699617.1	A0A6I8TRN6.1.A	100	96.32	0.56
GABA Receptor	AAA68961.1	A0A6I8TAT1.1.A	96.62	91.19	-1.44
TRP Channel	AAEL005437	A0A182GH52.1.A	95.58	93.12	-1.99
Octopamine Receptor	XP_021692997.1	A0A6I8T9G8.1.A	100	90.39	-1.95
Odorant Binding Protein	AaegOBP1	3K1E	100	99.15	0.39
Odorant Receptor - AaOr31	AAEL013217	A0A1S4FYV0.1.A	98.95	98.68	-1.22

References

1. Possel, R.D.; Souza, T.P.; Oliveira, D.M.; Ferreira, M.O.; Dias, D.P.; Moraes, G.K.A.; Ferraz, L.F.; Ferreira, T.P.; Fernandes, A.C.; Didonet, J. Larvicide and repellent activity of *Hypenia irregularis* (Benth.) Harley in the alternative control of mosquito *Aedes aegypti*. *J. Med. Plants Res.* **2020**, *14*, 535-543, doi:https://doi.org/10.5897/JMPR2019.6777.
2. Dalcin, M.S.; Dias, B.L.; Viteri Jumbo, L.O.; Oliveira, A.C.S.S.; Araújo, S.H.C.; Moura, W.S.; Mourão, D.S.C.; Ferreira, T.P.S.; Campos, F.S.; Cangussu, A.S.R.; et al. Potential action mechanism and inhibition efficacy of *Morinda citrifolia* essential oil and octanoic acid against *Stagonosporopsis cucurbitacearum* infestations. *Molecules* **2022**, *27*, 5173, doi:https://doi.org/10.3390/molecules27165173.
3. Coelho-de-Souza, A.; Alves-Soares, R.; Oliveira, H.; Gomes-Vasconcelos, Y.; Souza, P.; Santos-Nascimento, T.; Oliveira, K.; Diniz, L.; Guimarães-Pereira, J.; Leal-Cardoso, J. The essential oil of *Hyptis crenata* Pohl ex Benth. presents an antiedematogenic effect in mice. *Braz. J. Med. Biol. Res* **2021**, *54*, e9422, doi:http://dx.doi.org/10.1590/1414-431X20209422.
4. Mourão, D.D.S.C.; Ferreira de Souza Pereira, T.; Souza, D.J.d.; Chagas Júnior, A.F.; Dalcin, M.S.; Veloso, R.A.; Leão, E.U.; Santos, G.R.d. Essential Oil of *Cymbopogon citratus* on the Control of the Curvularia Leaf Spot Disease on Maize. *Medicines* **2017**, *4*, 62, doi:https://doi.org/10.3390/medicines4030062.
